# Association between Physical and Motor Fitness with Cognition in Children

**DOI:** 10.3390/medicina55010007

**Published:** 2019-01-04

**Authors:** Akbar Moradi, Esmaeil Sadri Damirchi, Mohammad Narimani, Samad Esmaeilzadeh, Inga Dziembowska, Liane B. Azevedo, Wagner Luiz do Prado

**Affiliations:** 1 Islamic Azad University Science and Research Branch, Tehran 1477893855, Iran; akbar33029@gmail.com; 2 Department of Counseling, University of Mohaghegh Ardabili, Ardabil 5619911367, Iran; E.Sadri@uma.ac.ir; 3 Department of Psychology, University of Mohaghegh Ardabili, Ardabil 5619911367, Iran; narimani@uma.ac.ir; 4 University of Mohaghegh Ardabili, Ardabil 5619911367, Iran; 5 Department of Pathophysiology, Faculty of Pharmacy, Collegium Medicum in Bydgoszcz, Nicolaus Copernicus University in Toruń, 85-094 Bydgoszcz, Poland; i.dziembowska@cm.umk.pl; 6 School of Health and Social Care, Teesside University, Middlesbrough TS1 3BA, UK; L.Azevedo@tees.ac.uk; 7 Human Movement Sciences and Rehabilitation Graduation Program, Federal University of São Paulo, São Paulo 11030-020, Brazil; wagner.prado@unifesp.br

**Keywords:** agility, cognition, fitness, inhibitory control, reaction time, schoolboys

## Abstract

*Background and objective:* There is an increased interest in exploring the association between fitness components with cognitive development in children in recent years. One of the scopes is to find the best exercise prescription to enhance health and cognition. Most of the studies so far have focused on cardiorespiratory fitness with little evidence on other fitness components. The present study aimed to explore the association between physical fitness (PF) and motor fitness (MF) with cognitive performance in children. *Methods:* Two hundred and six schoolboys (11.0 ± 0.8 y) underwent a battery of tests to measure information processing speed (i.e., simple and choice reaction time) and inhibitory control (i.e., Simon task). PF components (i.e., flexibility, muscular strength, and endurance) and MF components (speed and agility) were measured. *Results:* Multiple linear regression analysis adjusted for potential confounders (i.e., age, socioeconomic status, %fat and physical activity) revealed no relationship between flexibility, speed, muscular strength, and endurance with either information processing tasks or inhibitory control tasks. However, a positive association was observed between agility with both congruent reaction time and incongruent reaction time. *Conclusions:* No relationship was observed between the underlying fitness components with either information processing or inhibitory control. However, an association was observed between agility with inhibitory control.

## 1. Introduction

The association between fitness components and cognitive development in children has been frequently studied in recent years [1,2,3,4]. The research focus is now shifting to find the most appropriate exercise prescription to enhance health and cognition [1,2]. Fitness is a physiological state of well-being that decreases the risk of hypokinetic disease (i.e., diseases related with physical inactivity and disuse) and is a basis for participation in sports and health that enables an individual to carry out the tasks of daily living [4]. Cardiorespiratory fitness (CRF) has been recognized as one of the most important components of fitness. It has been associated negatively with a clustering of metabolic risk factors in children [5]. On the other hand, several studies have shown a significant association between CRF with cognitive function [2,6,7] and academic achievements in children [3,7]. However, fitness is a multifaceted concept including physical fitness (PF) and motor fitness (MF). PF comprises a set of measurable health- and skill-related attributes, such as: CRF, muscular strength and endurance, body composition, and flexibility, whereas MF includes: Speed, agility, coordination, balance, and power [8]. However, most of the studies underlying the association between fitness and cognition have limited their analysis to CRF, and there are limited data supporting the hypothesis that other fitness components may also influence brain functions [1,9,10,11]. These recent studies have suggested that exploring other components may help with the design of a better exercise program which might improve cognitive function further if compared to CRF-oriented programs only [1,2,9,11,12]. For instance, it has been reported that not all forms of aerobic exercise benefit executive function equally and cognitively engaging exercise (e.g., participation in group activities or sports that require complex cognition) seems to have a larger impact than non-engaging exercise (e.g., simple repetitive aerobic exercise) on children’s executive function [2].

The results regarding the association between different fitness and cognitive functions in children are inconsistent and scarce for some cognitive functions [1,4]. Further evidence might help to clarify the existence and strength of this association [4].

Fernandes et al. [13] explored the association between motor skills (motor coordination, agility), cognitive function (Stroop test and Wechsler Intelligence Scale), and school performance (academic achievement test) in a sample of 8–14-year-old students. They observed that visual selective attention and visual motor coordination influenced academic achievement and cognitive function. However, the same association was not observed for agility. The authors suggested the need to further explore the association between physical skills and different aspects of cognition. Likewise, Moradi and Esmaeilzadeh [14] studied a sample of Iranian schoolboys and observed that agility (but not speed of movement) was significantly associated with information processing speed. In another study with a sample of young Iranian males (19–24 years) [15], explosive strength was a significant predictor of both inhibitory control and information processing; however, aerobic fitness was only a significant predictor of inhibitory control. Finally, in a recent systematic review, Donnelly et al. [4] reported that although there is a positive relationship between physical activity (PA), fitness, academic achievement, and cognition, the findings are inconsistent and there is a need for further research to explore this relationship.

Inhibitory process is the ability of the cognitive process to prevent dominant or habitual responses to stimuli in order to select a more appropriate response or behavior and, in this regard, can interfere with the completion of cognitive, motor or socioemotional goals [16]. The processes are critical in everyday life to allow to complete actions successfully (e.g., avoiding impulsive behavior, stopping at traffic lights) [17].

On the other hand, information processing speed is a cognitive ability that measures how quickly a person can perform cognitive tasks, especially simple cognitive tests, such as simple and choice reaction time [18]. Furthermore, the tasks are shown as an overall evaluation of cognitive mechanisms, which are broadly applied to support the motor and cognitive processes, and fluent execution of perception [19]. Same as inhibition, attention, and working memory, information processing speed is considered a critical cognitive resource and correlated with performance in various cognitive domains [18,20].

Although information processing speed and inhibitory control of cognitive tasks play a critical role in our daily lives [16,21], to the best of our knowledge, there is little and inconsistent evidence investigating the association between these variables and various fitness components in children [1]. The aim of the present study was to explore the association between different components of fitness (e.g., muscular strength, endurance, speed, and agility) with inhibitory control and information processing speed tasks in a homogeneous sample of primary schoolboys adjusted for potential confounders such as age, SES, adiposity, and PA.

## 2. Methods

### 2.1. Participants

**Figure 1:**
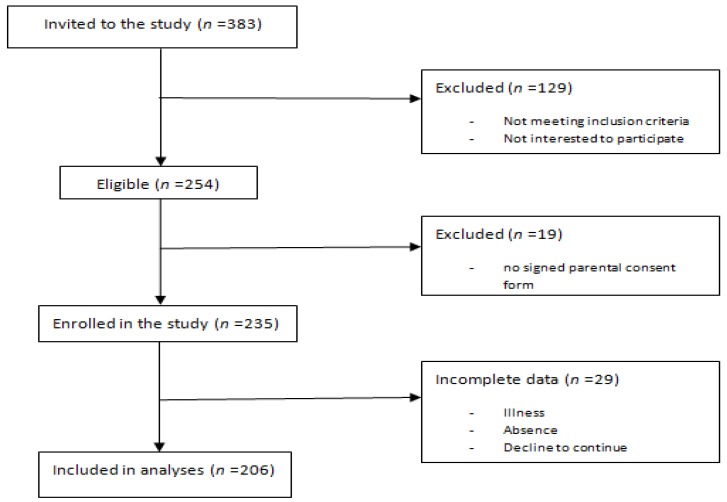
Experimental design.

This cross-sectional study was conducted in 10–12-year-old boys in the center of Ardabil Province, North West of Iran, during 2015 and 2016. Due to sociocultural reasons, only boys were included in the sample. Three schools were selected randomly from a list of public schools. Fifteen classes were then randomly selected from a list of fourth, fifth, and sixth grades and 383 students within these classes were invited to participate. Participants were excluded if they presented musculoskeletal problems or chronic diseases, were using medications or were not interested in participating (129 participants were excluded). Participants provided verbal consent and presented written consent by their parents. Most of the students were interested in participating in the study and completing the tests (participation rate = 84%), except for nineteen participants who did not present the signed written consent and therefore were not included. Finally, data from twenty-nine participants who did not complete all measurements were excluded from the analyses (Figure 1).

Participants ages were obtained from the school register. All measurements were conducted at the schools during physical education classes. The study was conducted following the Declaration of Helsinki, and the protocol was approved by the Ethics Committee of Mohaghegh Ardabili University (Consent No 001/2015 from 25 September 2015).

### 2.2. Anthropometry

Body mass was measured with minimal clothing and without shoes using an electronic scale (Type SECA 861) and measured to the nearest 0.1 kg. Height was measured barefoot in the Frankfurt horizontal plane with a telescopic height measuring instrument (Type SECA 225) to the nearest 1 mm. Both height and weight were measured twice at the first visit. If there were differences (≥±0.1 kg) between measurements, a third measurement was performed, and the average was calculated and reported.

### 2.3. Physical and Motor Fitness Tests

Fitness tests included both PF and MF components as follows: Static muscular strength (i.e., grip strength test), dynamic muscular endurance (i.e., sit-ups test and modified pull-ups), flexibility (i.e., sit and reach test), agility (i.e., 4 × 10 m shuttle run test), and movement speed (i.e., 30-m sprint test). Before measuring the tests, all students were first familiarized with the procedures. The students were instructed not to perform vigorous PA on the same day and one day before the fitness assessment. PF tests (i.e., flexibility, muscular strength, and endurance) were performed at the first week (when the students attended the physical education lesson), while MF tests (speed and agility) were measured at the following week. Measuring procedures for PF and MF components were as follows:

#### 2.3.1. Physical Fitness

Static muscular strength (SMS) was performed with a standard calibrated digital hand dynamometer (TKK, Model 5401; Takei, Tokyo, Japan) that was calibrated periodically according to the manufacturer’s manual. Schoolboys were tested in a sitting position with shoulder adducted, elbow at 90° flexion, and wrist and forearm in a neutral position. They were instructed to squeeze the dynamometer as forcefully as possible and to sustain the effort for 5 s. The test was repeated twice for each hand with a 1-min break between trials and alternating hands to minimize the fatigue effect. The best score for each hand was recorded in kilograms. Hand grip strength has been validated for measuring SMS and been associated with lower and upper body strength (*r* = 0.736 to 0.890) [22].

Dynamic muscular endurance (DME): Sit-ups and modified pull-ups tests were used for measuring DME. Bent-knee sit-ups as a valid and reliable (*r* = 0.62 to 0.93) test measured the endurance of the abdominal muscles. It was recorded by the maximum number of sit-ups achieved in one minute [23,24]. Using modified pull-ups as a valid and reliable (*r* = 0.89 to 0.91) test for measuring upper body muscular endurance, participants were positioned on their backs with shoulders directly under a bar that was set one to two inches above their reach. Participants started in the lower position with arms and legs straight and with their heels touching the floor and then had to pull themselves up and down rhythmically [23,24].

Mean of T scores of both the tests (sit-ups and pull-ups) provided the DME result.

Flexibility: A sit and reach test was used for measuring flexibility as a valid and reliable *(r* = 0.94 to 0.97) test for measuring the flexibility of the hamstrings, buttocks, and lower back [23,24]. For this test, the participants sat on the ground with their legs straight against a standard reach box where 23 cm was marked at the level of participants’ feet. Participants were instructed to smoothly reach forward and sustain themselves in the extreme reach position for two seconds.

#### 2.3.2. Motor Fitness

Movement speed: A 30-m sprint test was used for this purpose. This test involves running a single maximum sprint over 30 m and was used for measuring the speed of movement [14].

Agility: The 4 × 10 m shuttle run test as a valid test and with acceptable reliability [25] was used for agility. The test measured agility, speed of movement, and coordination. On command, the participants ran from a start line across a court to pick up one block and then returned to put it behind the starting line and ran back again to pick up the second block and ran back again across the starting line. A hand-held stopwatch was used for measuring time to the nearest 0.01 s (Joerex, ST4610-2, Guangdong, China). For the grip strength, sit and reach, speed of movement, and agility tests, the best value of two to three consecutive trials was used for the statistical analysis.

### 2.4. Cognitive Tests

All participants were instructed to avoid caffeine drinks 3–4 h and high-intensity PA twenty-four hours before the tests. All the cognitive tests were performed in an empty room and were measured in different days from the fitness tests. Before starting the cognitive tests, participants were first familiarized with the test procedures. Participants were allowed rest breaks (5 min) between each test to prevent mental fatigue. The following cognitive type tests were used: 1. Information processing speed: Simple reaction time (SRT) and 4-choice reaction time (4-CRT) and 2. Inhibitory control: Simon task. These are detailed below.

#### 2.4.1. Information Processing Speed

Simple reaction time (SRT) and 4-choice reaction time (4-CRT): Participants performed the Deary–Liewald computer-based reaction time (RT), including the SRT and 4-CRT tasks, which has been previously validated [21]. The Deary–Liewald computer-based RT is a free program and can be downloaded at the following address: https://www.ccace.ed.ac.uk/news-events/latest/reaction-time-task-new. The SRT task involved eight practice trials and twenty test trials. The participants were requested to respond (press spacebar) to a single stimulus as quickly as possible (response range from 150 ms to1500 ms; inter stimulus interval from 1000 ms to 3000 ms). The 4-CRT task involved eight practice trials followed by 40 test trials. In the 4-CRT, the participant was asked to respond to a stimulus as quickly as possible by pressing the correct key (choice of up, down, left, right arrow keys) that corresponded to the correct response to four different stimuli (response range from 200 ms–1500 ms; inter stimulus interval from 1000 ms–3000 ms). Response accuracy for the 4-CRT task was 0.89.

#### 2.4.2. Inhibitory Control

For measuring inhibitory control, the Simon task [26] was chosen through the Cognitive Psychology Online Laboratory (CogLab) program [27]. Coglab is a computer training program developed to enhance the various dimensions of cognition (i.e., attention, perception, sensory memory, etc.). Simon task is a valid test to measure cognitive control (e.g., attention) through the ability to inhibit incorrect response impulses [26]. The procedure of this test includes the placement of a white, small square in the center of the display to be maintained throughout the trials (*n* = 100) as a gaze fixation point. The participant was requested to respond as accurately and quickly as possible to the color of an oval delivered either to the left or the right of the white gaze-fixation square by pressing the appropriate left or right arrow response key (stimuli were presented in random order; inter stimulus interval from 500 to 1500 ms). The task included two equiprobable trial types as follows: The congruent trials (ConRT), in which the spatial location of the stimulus corresponded to the task-relevant aspect of the stimulus (for example right stimulus/right response), and the incongruent trials (InconRT), in which the spatial location of the stimulus corresponded to the opposite spatial location of the response (for example, right stimulus/left response). A difference score was calculated to measure inhibition (∆ Simon: Time on IncoRT minus time on ConRT). The program was designed to save only true responses, and thus, response accuracy for the Simon task was 1.0 [27].

### 2.5. Possible Confounders

Some possible confounders were measured and used in the analysis. Percentage of body fat is stated as a more reliable overall obesity index than BMI and in this study was estimated using the Slaughter et al. [28] equation. Triceps and calf skinfold were measured at the right side of the body, and the average of three measures was calculated for each site using a Lange caliper. Socioeconomic status (SES) was computed from parents’ occupational and educational status as explained in the previous study [14]. PA level was estimated using the PA questionnaire for children (PAQ-C), which measured the PA behaviors of the boys at different places and times (i.e., weekend, after school, during school, recess, etc.) during the previous seven days. PAQ-C has been previously validated and applied in Iranian children [29,30]. Participants were requested to fill out the questionnaire under their parents’ supervision. Scoring of the PAQ-C was based on a five-point Likert scale, with an overall PA score that derived from an average of each scored item and a greater score indicating higher levels of PA.

### 2.6. Statistical Analyses

Descriptive statistics were presented for all variables. All data were checked for normality using the Kolmogorov–Smirnov test. Pearson correlations were conducted to verify the association between the variables, while multiple linear regression analyses using the Enter method were conducted between cognitive and fitness tests by adjusting for age, %fat, SES, and PA. To interpret the Pearson correlations, the following Cohen ranges were used: 0.1 < |*r*| <0.3 small association, 0.3 < |*r*| <0.5 moderate association, and 0.5 < |*r*| strong association [31]. Furthermore, to interpret the regression coefficient Cohen’s F^2^ [31], the following range was used: 0.02 = small, 0.15 = medium, and 0.35 = large. Assuming a power of 0.977 and an alpha of 0.01, a sample of 206 individuals was estimated to be required to explore a medium effect size (Fsq = 0.15) for analyses including five predictors and a single dependent variable in each model [32]. Therefore, the sample of the present study had strong power to explore medium to large effect sizes. All analyses were performed using SPSS v.21.0 (SPSS Inc., Chicago, IL, USA). Statistical significance was set at *p* < 0.05.

## 3. Results

### 3.1. General Characteristics of the Participants

Mean and standard deviation (SD) of age, height, weight, and %fat of boys (*n* = 206) were 11.0 ± 0.8 year; 146.4 ± 8.8 cm; 46.9 ± 12.7 kg; and 22.1 ± 7.3%; respectively. Mean, SD, and normality of participants’ physical status (including PA and fitness) and cognition are presented in Table 1.

Results suggest that variables such as agility, speed, SRT,4-CRT, ConRT, InconRT, and ∆ Simon did not present a normal distribution. However, normal distribution was achieved after transformation using a natural logarithm. Therefore, natural logarithm data of agility, speed, SRT,4-CRT, ConRT, InconRT, and ∆ Simon were used in the analyses (Pearson correlation and multiple regression analysis). Analysis of normal P–P plots of the residuals, histograms, and scatter plots of the residuals indicated that the assumption of homoscedasticity was met.

### 3.2. Pearson Correlation among the Study Variables

Pearson moment correlation (Table 2) showed weak associations between age with SRT (*r* = −0.18; *p* = 0.02) and ConRT (*r* = −0.28; *p* < 0.001) but moderate association with InconRT (*r* = −0.33; *p* < 0.001). A weak negative association was observed between PA and ∆ Simon (*r* = −0.18; *p* = 0.03). Similarly, there was a weak positive association between agility with ConRT (*r* = 0.21; *p*= 0.007) and InconRT (*r* = 0.15; *p* = 0.05). Fat percentage was moderately and negatively associated with PA (*r* = −0.45; *p* < 0.001) but was moderately and positively associated with agility (*r* = 0.45; *p* < 0.001). Fat percentage was weakly and negatively associated with flexibility (*r* = −0.25; *p* = 0.002) and positively and weakly associated with SMS (*r* = 0.18; *p* = 0.02). Fat percentage was strongly and negatively associated with DME (*r* = −0.60; *p* < 0.001) but was strongly and positively associated to run speed (*r* = 0.55; *p* < 0.001). A moderate association was also observed between PA with flexibility (*r* = 0.39; *p* < 0.001) and agility (*r* = −0.44; *p* < 0.001), but a strong association was observed between PA with DME (*r* = 0.55; *p* < 0.001) and run speed (*r* = −0.50; *p* < 0.001). No significant association was observed between PA and SMS (*r* = −0.05; *p* = 0.54). ∆ Simon was weakly and positively associated with InconRT (*r* = 0.21; *p* = 0.008) and weakly and negatively associated with PA (*r* = −0.17; *p* = 0.03).

### 3.3. Multiple Linear Regression Analysis between Cognitive Tasks with PF and MF Components

Multiple linear regression analysis (Table 3) after adjusting for possible confounders (i.e., age, %fat, SES, and PA) revealed no association between both the PF and MF components with any of the information processing tasks (i.e., SRT and CRT).

Multiple linear regression analysis indicated no significant association between both the PF and MF components with any of the inhibitory control tasks except for a significant positive association between agility with ConRT (Standardized β = 0.22; *p* = 0.01) and InconRT (Standardized β = 0.19; *p* = 0.03). The Cohen’s F^2^ effect size for the association between agility with ConRT was 0.13 and considered small. The effect size for the association between agility with InconRT was 0.16 and therefore a medium association.

No significant association was found between any of the PF and MF components with ∆ Simon.

## 4. Discussion

The present study aimed to explore the association between PF and MF with cognitive function in schoolboys. The results of the present study showed that agility as a component of MF was a significant predictor of inhibitory control but not information processing speed. None of the PF components (SMS, DME, and flexibility) or other measured MF components (i.e., speed) were predictors of either information processing speed or inhibitory control in the schoolboys. These findings are consistent with the literature [1,11,33].

To the best of our knowledge, the present study is one of few exploring the association between cognitive performance with both PF and MF in elementary schoolboys [1]. The existing evidence about the association between fitness and cognitive function in children is limited and inconsistent [1]. The inconsistency might be associated with the fact that variables have not been adjusted for important confounders, such as age, adiposity, socioeconomic status (SES), and PA. Other reasons might include small sample size, use of cumulative scores for fitness components or a large variation in the population (i.e., age ranges, gender) or outcome variables (i.e., cognitive functioning and fitness tests). For instance, Niederer et al. [33] observed that agility was independently associated with better performance both in working memory and attention in children after controlling for potential confounders, such as age and SES. Davis et al. [34] found a significant association between strength/agility with visual processing, memory, and crystallized intelligence (but not with fluid intelligence) among children. Roebers and Kauer [35] have found no association between executive functioning tasks (e.g., inhibitory control) and motor skills (i.e., body coordination, postural flexibility and moving sideways) among 6–9-year-old children. Similar findings were observed in another study [36], where strength, flexibility. and agility were not associated with fluid intelligence in 10- and 14-year-old children, although a significant association was observed between flexibility and fluid intelligence in 12-year-old boys. Moradi and Esmaeilzadeh [14] observed that only agility (but not speed) was significantly associated with information processing speed (measured using clinical RT) in children. However, in another study with young males aged 19–24 years, Esmaeilzadeh et al. [15] found no association between agility and either information processing speed or inhibitory control. Katic and Bala [37], studying a sample of prepubertal and pubertal girls and using a variety of fitness tests, observed that cognitive functioning was associated with agility. Moreover, in pubertal girls, cognitive functioning was also associated with other fitness components, such as jumping, sprinting, agility, and trunk strength. Most recently, Hartman et al. [38] studied a sample of children with intellectual disabilities or borderline intellectual disabilities and observed that skill-related PF (e.g., agility and coordination) was significantly associated with inhibition and cognitive flexibility. However, interestingly, they found no significant association between aerobic fitness and executive function.

Our findings are consistent with the literature, which shows that motor skills activities which have higher cognitive demand are more likely to be associated with cognitive tasks [1,2]. Therefore, it is possible that some components of fitness, such as agility, could be a better predictor of cognition, perhaps even better than aerobic fitness in children [38], and can be included as a potential component of PA training programs for enhancing cognition [2,11,39,40]. In this regard, it has been recently argued that the volume of engagement in executive function (e.g., using group activities or sports that require complex cognition) during exercise could be a potential factor on cognitive function in children than non-engaging exercise (e.g., simple repetitive aerobic exercise) [2]. Therefore, there is a need to design PA programs which require constant shifts in motor response and attention (i.e., cognitive flexibility) with the aim of stimulating prefrontal lobe functioning and therefore increasing children’s inhibitory ability [39,40]. In this regard, Lennemann et al. [11] explored the effect of agility training vs. traditional physical training on cognition in a sample of young people. They observed that agility training promoted significant improvements not only in CRF and physical agility but also cognitive skills, such as working memory and sustained attention, compared with traditional physical training.

### Limitations

The present study has some limitations. The cross-sectional design avoids the inference of causality and longitudinal studies are needed to clarify the direction of the association. Due to sociocultural reasons, we were unable to include girls in the sample. Therefore, the results cannot be generalized for girls. Likewise, CRF was not measured. Although previous studies have extensively explored the association between CRF and cognition, we have not measured CRF in this study, which could have extended previous findings [2,4,7]. Another limitation is that other measures of cognition (e.g., working memory, long-term memory, task-switching) were not assessed and an association might exist for one or more of these other components.

Nonetheless, the present study has some strengths, including the use of linear models to assess the relationship between the variables and controlling for covariates. Likewise, most studies on this topic have been performed on populations in the United States and Europe, and this is the first study developed with a sample of boys from the Middle East.

## 5. Conclusions

In summary, PF and MF components appear not to be associated with cognitive tasks in boys, except for the positive association between agility and inhibitory control, which was considered small and medium for ConRT and InconRT, respectively. Interventional agility training programs in comparison to other training modalities (e.g., aerobic training, flexibility training) are needed to establish whether agility programs can enhance cognition in children.

## Figures and Tables

**Table 1:** General characteristics of participants (*n* = 206) and normality of the variables.

		Mean (SD)	Before Transformation	After Transformation
			Kolmogorov–Smirnov Test (*p*)	Kolmogorov–Smirnov Test (*p*)
PA (score)		2.5 (1.9)	*0.18*	
PF components	Pull-up (reps)	8.9 (6.3)	*0.08*	
	Sit-ups (reps)	25.2 (11.7)	0.20	
	DME (T score)	50.0 (0.7)	0.20	
	Sit and Reach (cm)	23.1 (7.6)	*0.20*	
	Grip strength (kg)	18.2 (5.1)	*0.20*	
MF components	4 × 10 m Agility (s)	12.8 (0.9)	*0.04*	*0.12*
	30 m Run Speed (s)	6.5 (0.6)	*0.05*	0.19
Information processing speed	SRT (ms)	331.0 (53.6)	0.04	*0.20*
	4-CRT (ms)	535.0 (92.1)	*0.01*	*0.20*
Inhibitory control	ConRT (ms)	646.7 (128.6)	*<0.01*	*0.14*
	InconRT (ms)	710.4 (131.8)	*<0.01*	*0.15*
	∆ Simon (ms)	63.7 (51.3)	*<0.01*	*0.10*

DME: Dynamic muscular endurance; MF: Motor fitness; PF: Physical fitness; PA: Physical activity; 4-CRT: 4-choice reaction time; ConRT: Reaction time for the congruent task; InconRT: Reaction time for the incongruent task; SRT: Simple reaction time; ∆ Simon: Simon incongruent minus congruent.

**Table 2:** Correlation among the study variables.

	SES	%fat	PA	DME	Flexibility	SMS	Agility	Speed	SRT	4-CRT	ConRT	InconRT	∆ Simon
Age	0.01	−0.08	−0.04	0.02	−0.10	0.16 *	−0.08	−0.26 **	−0.18 *	−0.02	−0.28 **	−0.33 **	−0.13
SES		0.03	0.14	0.03	-0.10	0.10	0.07	0.03	−0.08	0.07	0.06	0.03	−0.06
%fat			−0.45 **	−0.60 **	−0.25 **	0.18 *	0.45 **	0.55 **	−0.01	0.05	0.04	−0.02	−0.04
PA				0.55 **	0.39 **	−0.05	−0.44 **	−0.50 **	0.08	0.05	−0.03	0.04	−0.17 *
DME					0.42 **	0.17 *	−0.61 **	−0.63 **	-0.03	−0.01	−0.07	−0.01	0.01
Flexibility						0.11	−0.38 **	−0.23 **	0.02	−0.11	−0.03	−0.01	0.05
SMS							−0.16 *	−0.49 **	−0.08	0.02	0.01	0.01	0.03
Agility								0.73 **	0.05	0.09	0.21**	0.15 *	0.03
Speed									0.09	0.02	0.14	0.09	−0.04
SRT										0.39 **	0.34 **	0.32 **	0.01
4-CRT											0.59 **	0.57 **	0.02
ConRT												0.90 **	−0.10
InconRT													0.21 **

DME: Dynamic muscular endurance; 4-CRT: 4-choice reaction time; ConRT: Reaction time for the congruent task; InconRT: Reaction time for the incongruent task; PA: Physical activity; SES: Socioeconomic status; SRT: Simple reaction time; ∆ Simon: Simon incongruent RT minus congruent RT; SMS: Static muscular strength. Natural logarithm data of agility, speed, SRT,4-CRT, ConRT, InconRT, and ∆ Simon were used in the analysis. * significant at <0.05; ** significant at <0.01.

**Table 3:** Multiple linear regression analysis between cognitive tasks with PF and MF components.

		Tolerance	VIF	t	R^2^	Standardized β	*p*	95% CI
SRT	DME	0.54	1.85	0.02	0.050	0.002	0.98	−0.18 to 0.185
	Flexibility	0.83	1.20	0.27	0.050	0.02	0.78	−0.06 to 0.08
	SMS	0.68	1.47	−0.88	0.053	0.03	0.76	−0.03 to 0.04
	Agility	0.70	1.42	0.26	0.050	0.024	0.79	−0.33 to 0.43
	Speed	0.55	1.82	0.56	0.051	0.06	0.58	−0.24 to 0.42
4-CRT	DME	0.54	1.87	0.30	0.013	0.03	0.76	−0.17 to 0.23
	Flexibility	0.83	1.20	−1.19	0.021	−0.10	0.23	−0.12 to 0.03
	SMS	0.68	1.48	0.09	0.012	0.01	0.93	−0.03 to 0.04
	Agility	0.72	1.40	1.17	0.021	0.11	0. 24	−0.17 to 0.65
	Speed	0.55	1.80	0.19	0.013	0.02	0.85	−0.33 to 0.40
ConRT	DME	0.53	1.87	−0.71	0.086	−0.07	0.48	−0.30 to 0.14
	Flexibility	0.83	1.21	−0.02	0.083	−0.001	0.95	−0.08 to 0.08
	SMS	0.68	1.50	−0.17	0.081	−0.02	0.86	−0.04 to 0.04
	Agility	0.71	1.40	2.50	0.117	0.22	0.01	0.11 to 0.99
	Speed	0.55	1.81	0.71	0.086	0.07	0.48	−0.25 to 0.53
InconRT	DME	0.53	1.87	−0.37	0.111	−0.04	0.71	−0.24 to 0.16
	Flexibility	0.83	1.21	0.05	0.110	0.004	0.96	−0.07 to 0.08
	SMS	0.68	1.48	0.15	0.111	0.01	0.87	−0.05 to 0.04
	Agility	0.71	1.41	2.23	0.137	0.19	0.03	0.05 to 0.86
	Speed	0.55	1.81	0.37	0.111	0.04	0.71	−0.29 to 0.42
∆ Simon	DME	0.55	1.80	−0.89	0.045	−0.10	0.37	−1.64to 0.62
	Flexibility	0.85	1.20	−0.34	0.041	−0.03	0.73	−0.48 to 0.34
	SMS	0.69	1.45	−0.53	0.042	−0.05	0.60	−0.26 to 0.15
	Agility	0.74	1.35	1.17	0.049	0.11	0.24	−0.93 to 3.62
	Speed	0.57	1.74	1.28	0.050	0.13	0.20	−0.68 to 3.19

SRT: Simple reaction time; 4-CRT: 4-choice reaction time; ConRT: Reaction time for congruent task; InconRT: Reaction time for incongruent task; ∆ Simon: Simon incongruent minus congruent; SMS: Static muscular strength; DME: Dynamic muscular endurance. DME was computed from a mean of T score of the sit-ups and pull-ups tests. Natural logarithm data of agility, speed, SRT,4-CRT, ConRT, InconRT, and ∆ Simon were used in the model.
